# Refocusing cancer supportive care: a framework for integrated cancer care

**DOI:** 10.1007/s00520-022-07501-9

**Published:** 2022-12-14

**Authors:** Meinir Krishnasamy, Amelia Hyatt, Holly Chung, Karla Gough, Margaret Fitch

**Affiliations:** 1 Academic Nursing Unit, Peter MacCallum Cancer Centre, 305 Grattan Street Parkville, Melbourne, Australia; 2 Health Services Research Group, Peter MacCallum Cancer Centre, Melbourne, Australia; 3 Sir Peter MacCallum Department of Oncology, The University of Melbourne, Victoria, 3010 Australia; 4 Victorian Comprehensive Cancer Centre Alliance, Victoria, 3010 Australia; 5 Department of Nursing, Faculty of Medicine, Dentistry, and Health Sciences, The University of Melbourne, Melbourne, 3052 Australia; 6 Bloomberg Faculty of Nursing, University of Toronto, Toronto, Canada

**Keywords:** Cancer, Supportive care, Conceptual framework, Definitions, Health system

## Abstract

**Objective:**

Cancer supportive care comprises an integrative field of multidisciplinary services necessary for people affected by cancer to manage the impact of their disease and treatment and achieve optimal health outcomes. The concept of supportive care, largely driven by Margaret Fitch’s seminal supportive care framework, was developed with the intent to provide health service planners with a conceptual platform to plan and deliver services. However, over time, this concept has been eroded, impacting implementation and practice of supportive care. This study therefore aimed to examine expert contemporary views of supportive care with the view to refocusing the definition and conceptual framework of cancer supportive care to enhance relevance to present-day cancer care.

**Methods:**

A two-round online modified reactive Delphi survey was employed to achieve consensus regarding terminology to develop a contemporary conceptual framework. A listing of relevant cancer supportive care terms identified through a scoping review were presented for assessment by experts. Terms that achieved ≥ 75% expert agreement as ‘necessary’ were then assessed using Theory of Change (ToC) to develop consensus statements and a conceptual framework.

**Results:**

A total of 55 experts in cancer control with experience in developing, advising on, delivering, or receiving supportive care in cancer took part in the Delphi surveys. Expert consensus assessed current terminology via Delphi round 1, with 124 terms deemed relevant and ‘necessary’ per pre-specified criteria. ToC was applied to consensus terms to develop three key statements of definition, and a comprehensive conceptual framework, which were presented for expert consensus review in Delphi round 2.

**Conclusion:**

Finalised definitions and conceptual framework are strongly aligned with relevant international policy and advocacy documents, and strengthen focus on early identification, timely intervention, multidisciplinary collaboration, and end-to-end, cross-sector, cancer supportive care.

## Introduction

Due to the rapid evolution of modern anti-cancer treatment, cancer is now considered a chronic disease [1, 2]. Novel treatments deliver longer survival, but often with a range of acute toxicities and long-term side effects which negatively impact quality of life and necessitate ongoing health service use [2, 3]. Furthermore, appreciation of the far ranging psychosocial consequences of a cancer diagnosis, such as financial toxicity or fear of cancer recurrence, has become much more apparent [4].

As an integrated field of multidisciplinary interventions, cancer supportive care comprises services necessary for people affected by cancer to manage the demands of disease and treatment [1]. The concept of supportive care and recognition of its contribution to comprehensive cancer services has been largely driven by Margaret Fitch’s seminal supportive care framework, published almost 20 years ago [5]. This person-centred framework highlights seven key domains which need to be routinely and iteratively assessed and supported across the entire cancer pathway to deliver optimal patient experience and outcomes of care: informational, emotional, practical, physical, psychological, social, and spiritual [5].

The original intent of the framework was to provide health service planners with a conceptual platform to plan and deliver services. Importantly, the framework aimed to direct attention to the potential for supportive care to be required at all facets of the cancer journey: during screening, diagnosis, treatment, and follow-up cancer care [5]. Over time, numerous definitions of supportive care have arisen [6–8] resulting in a lack of consensus regarding the concept, which has impacted its application and availability across different care settings [1, 9, 10]. Initially, intended to be conceptualised as an approach to care delivery impacting the totality of a patient’s experience and outcomes, supportive care has become synonymous with separate or disparate clinical services and interventions targeting disaggregated needs [11, 12]. As a consequence, component elements of cancer supportive care have received differing levels of attention, prioritisation, and funding resulting in services which are highly fragmented, sporadically implemented, and poorly evaluated [13–15].

Erosion of the underlying principle of totality of experience and care as articulated in Fitch’s conceptual framework for supportive care appears to have arisen as a consequence of efforts to research and/or implement supportive care as a series of discreet interventions, delivered at static time-points, in an attempt to demonstrate impact on health outcomes [10]. Much of the research undertaken since the publication of the framework has presented descriptive reports of unmet needs across heterogeneous cohorts of cancer patients at all stages of their illness pathway, or has attempted to demonstrate efficacy of discrete interventions targeted at specific domains of need. This disaggregated approach to Fitch’s original conceptualisation has resulted in a body of evidence that has largely failed to generate robust data of its benefit at patient or system levels. In turn, this has damaged clinicians and health service administrators’ perceptions of the value of investing in comprehensive supportive care as a fundamental component of quality cancer care.

Given the remarkable changes in cancer treatments and care since the publication of Fitch’s original Supportive Care Framework, it seemed opportune to re-visit and, if appropriate, refocus the definition and framework of cancer supportive care. A key characteristic of quality, person-centred care, is that regardless of diagnosis, or the severity or nature of need, all individuals experience the same level of access and ability to have their healthcare needs fulfilled [11]. To that end, quality cancer supportive care must be conceptualised and measured by its impact on patient experiences and outcomes, rather than as discrete clinical services and interventions [12, 16]. Influenced by a focus on value-based healthcare, where value is defined as outcomes that matter to patients [17], this study set out to examine contemporary views of supportive care. The objective was to establish consensus definition statements for cancer supportive care using a modified Delphi process [18] and, using these statements, inform the development of a conceptual framework to refresh the concept of cancer supportive care, relevant to present-day cancer care [19].

## Methods

A two-round online modified reactive Delphi was employed to identify consensus terms for supportive care definitions and deliver a contemporary conceptual framework for supportive care, using Theory of Change (ToC) [19]. This study was reviewed and approved by the University of Melbourne HREC (approval no: 1955021.1).

### Advisory group

An advisory group comprising key national stakeholders in supportive care was established to provide overarching guidance and input into the Delphi process. Members comprised: policy makers, clinicians, senior academics, cancer non-government organisation leaders, and consumer advocates.

### Design

This study utilised the Delphi technique: a structured, iterative process designed to facilitate expert contributions via sequential survey rounds to establish consensus on a particular topic or issue [20]. Specifically, a reactive modified Delphi approach was employed, by which the initial Delphi survey was developed through a comprehensive literature review of seminal supportive care definitions rather than open-text panel responses, followed by two, rather than three rounds of expert review and consensus-building. This approach was selected to acknowledge the importance and continuing relevance of published literature regarding supportive care, while maximising expert consensus in refreshing these statements.

#### Delphi round 1 development: scoping review

A scoping review was conducted to identify seminal published definitions of supportive care for adults affected by cancer. Identified definitions were analysed using qualitative content analysis to identify all unique terms used within definitions; similar terms were categorised [21]. All unique terms were then presented for consensus review in the Delphi round 1 survey.

#### Delphi round 2 development: Theory of Change

Using consensus terms and categories identified in Delphi round 1, refreshed supportive care statements of definition and a conceptual framework were developed and presented for expert review and consensus in the Delphi round 2 survey. ToC modelling was used to determine the person-, organisation- and system-level inputs and outputs necessary to deliver a contemporary supportive care framework true to the refreshed supportive care statements of definition [19].

### Delphi participants

Criteria used to select potential participants comprised experience of developing, advising on, delivering, or receiving supportive care in cancer. Potential participants working in clinical, research, policy, and quality roles in Australia, the UK, and Canada (countries recognised as leaders in cancer supportive care), and in consumer advocacy roles (which included patient and informal carers) were invited to participate. Special attention was made to extend invitations to those working in specialised areas of cancer supportive care, such as Aboriginal and Torres Strait Islander health and culturally and linguistically diverse (CALD) oncology care. Snowball recruitment techniques were applied at round 1, where participants were encouraged to send on the study invitation to colleagues whom they felt would also be appropriate to participate.

### Procedures

Invitations to participate were sent via email (either directly by the research team, or via snowball sampling). Those who were interested in taking part were directed to provide online informed consent. All participants who provided consent were emailed Delphi surveys for both rounds.

Delphi rounds 1 and 2 were designed and distributed to experts via REDCap online data collection and management software [22]. Surveys were sent approximately 3 months apart, with respondents allocated a 2-week window for completion (round 1: 20/08/2020–03/09/2020; round 2: 30/11/2020/08/12/2020). Two reminder emails were sent for each round. Each survey included clear description regarding Delphi development, aims, and purpose.

### Data collection

The Delphi round 1 survey included six demographic items and presented identified cancer supportive care terms organised by category. Each category included multiple terms; participants were requested to rate their agreement for inclusion for all terms they agreed were relevant, universal, and appropriate using a 7-point Likert scale (strongly agree, agree, somewhat agree, neither agree nor disagree, somewhat disagree, disagree, strongly disagree). Respondents were also invited to contribute their own terms to each category as they felt relevant.

In the Delphi round 2 survey, participants were asked to review the refreshed statements and conceptual framework and rate their agreement on a 7-point Likert scale against 11 statements regarding their design, proposed usage, and ability to support improved research and quality cancer supportive care. Respondents were invited to suggest changes to the wording for the refreshed statements or design of the conceptual framework and also provide additional comments regarding their agreement for or against the 11 statements.

### Data analysis

Participant agreement with terms presented in the Delphi round 1 survey was assessed against the following pre-specified consensus criteria: *Necessary*, ≥ 75% of experts felt that they agreed or strongly agreed with the inclusion of the term (consensus met); *Supplementary*, ≥ 60% experts felt that they agreed or strongly agreed with the inclusion of the term (considered meeting consensus only if ‘Necessary’ criteria is not met); *Unnecessary,* < 60% experts felt that they agreed or strongly agreed with the inclusion of the term (consensus not met). Additional terms suggested by respondents were analysed using content analysis, compared with existing terms to determine originality, and were retained or discarded as agreed by members of the project team.

Responses to Likert scale items presented in the Delphi round 2 survey were analysed descriptively using frequencies, range, means, and standard deviations. Interpretive description was used to analyse open-text items [23].

## Results

### Cancer supportive care definition refresh: terms and categories

A total of 12 papers were identified which contained seminal definitions of cancer supportive care (see Table 1 for list of papers).

**Table 1:** Review of literature containing seminal definitions of cancer supportive care

Author, year	Title
Boucher NA et al. 2017 [24]	Feasibility and acceptability of a best supportive care checklist among clinicians
Carrieri D et al., 2018 [9]	Supporting supportive care in cancer: the ethical importance of promoting a holistic conception of quality of life
Fitch M, 2008 [5]	Supportive care framework
Hui D, 2014 [10]	Definition of supportive care: does the semantic matter?
Klastersky J et al., 2016 [1]	Supportive/palliative care in cancer patients: quo vadis?
Koll T et al., 2016 [25]	Supportive care in older adults with cancer: across the continuum
Loeffen EA et al., 2017 [26]	The importance of evidence-based supportive care practice guidelines in childhood cancer—a plea for their development and implementation
Olver I, 2016 [3]	The importance of supportive care for patients with cancer
Olver I, 2022 [8]	Supportive care in cancer—a MASCC perspective
Rittenberg CN et al., 2010 [27]	An oral history of MASCC, its origin and development from MASCC’s beginnings to 2009
Victorian Government Department of Human Services, 2009 [28]	Providing optimal cancer care: supportive care policy for Victoria
Ward S et al., 2004 [29]	Improving supportive and palliative care for adults with cancer

Prior to the commencement of analysis, four definitions were excluded as terms used were not unique; that is, they were already covered in the remaining eight definitions. A total of 204 terms were identified; content analysis of these terms resulted in the development of the following 11 categories:

1. Individual health contexts related to cancer supportive care service delivery (example terms: cancer, cancer treatment)
2. Guiding actions for provision of cancer supportive care (example terms: assessment, screening, management, intervention, treatment)
3. Issues addressed by cancer supportive care services (example terms: side-effects, unmet needs, toxicity, adverse effects)
4. Who benefits from cancer supportive care services (example terms: patients, carers, family)
5. Cancer care continuum stages where cancer supportive care services should be available (example terms: diagnosis, treatment planning, survivorship, during palliative treatment, at relapse, at bereavement)
6. Overarching imperatives of cancer supportive care service delivery (example terms: evidence-based, comprehensive, integral, core component of care, timely, multi-specialty)
7. Who delivers cancer supportive care services (example terms: multi-disciplinary teams, nurses, general practitioners, psychologists, social workers, chaplains, specialist nurses)
8. Locations where cancer supportive care services are delivered (example terms: primary care, tertiary care, non-government organisations, palliative care unit, advocacy groups, community healthcare)
9. Achievement of cancer supportive care services (example terms: dignity, improved treatment outcomes, functional autonomy, empowerment, fewer post hospital complications)
10. Domains of cancer supportive care (example terms: social, physical, informational, psychological, practical, spiritual)
11. Specific clinical and psychosocial issues resolved by cancer supportive care services (example terms: cancer-related fatigue, hepatoxicity cachexia, mucositis, ascites, extravasation, alopecia, polypharmacy)

All terms and categories were presented to the Advisory Committee for review. A total of 23 items were deemed duplicates and were removed; however, all categories remained. Approved categories and terms were presented to experts in the Delphi round 1 survey.

### Delphi round 1 survey

A total of 61 people were invited to participate via direct and snowball email invitation. All those who provided consent (*n* = 55) completed the Delphi round 1 survey. Respondents were predominantly female (*n* = 47, 85% and were from a range of different healthcare sectors (see Table 2). A small proportion of overseas experts took part (7%, *n* = 4).

**Table 2:** Rounds 1 and 2 Delphi participants

		Round 1		Round 2	
Years worked in supportive care		*n* = *55*		*n* = *37*	
	*Mean, standard deviation*	14	11	16	12
	*Range*	2	45	2	45
Role category		***n***	***%***	***n***	***%***
	Clinician	21	38	14	38
	Researcher	6	11	6	16
	Policymaker	4	7	3	8
	Quality representative	12	22	4	11
	Consumer advocate	9	16	6	16
	Carer	2	4	2	5
	Other	1	2	1	3
	Missing	0	0	1	3
Gender					
	Male	7	13	5	14
	Female	47	85	31	84
	Missing	1	2	1	3
Postcode					
	Major city	39	71	22	59
	Inner regional	10	18	8	22
	Outer regional	2	4	1	3
	International	4	7	4	11
	Missing	0	0	2	5

### Building consensus

One hundred twenty-four of the 181 terms included in round 1 were identified as ‘necessary’ by participants and, therefore, met inclusion for consensus agreement. All items which met the ‘supplementary’ and ‘unnecessary’ criteria were discarded.

Terms identified as ‘necessary’ in category 10, domains of cancer supportive care, very closely aligned with those articulated by Margaret Fitch in her seminal work defining cancer supportive care [5]. Therefore, this category was removed from the ongoing process of consensus-building, because it was recognised as having continuing relevance. Category 11 comprised the listing of specific clinical and psychosocial issues resolved by cancer supportive care services. Category 11 was likewise removed due to systematic bias identified in the selection of terms rated ‘necessary’ by respondents. Specifically, medical terms were more likely to be excluded (e.g., ascites or extravasation) as opposed to terms more commonly understood by lay audiences (e.g., financial, sleep).

### Cancer supportive care definition refresh: ToC statements and conceptual framework

**Fig. 1:**
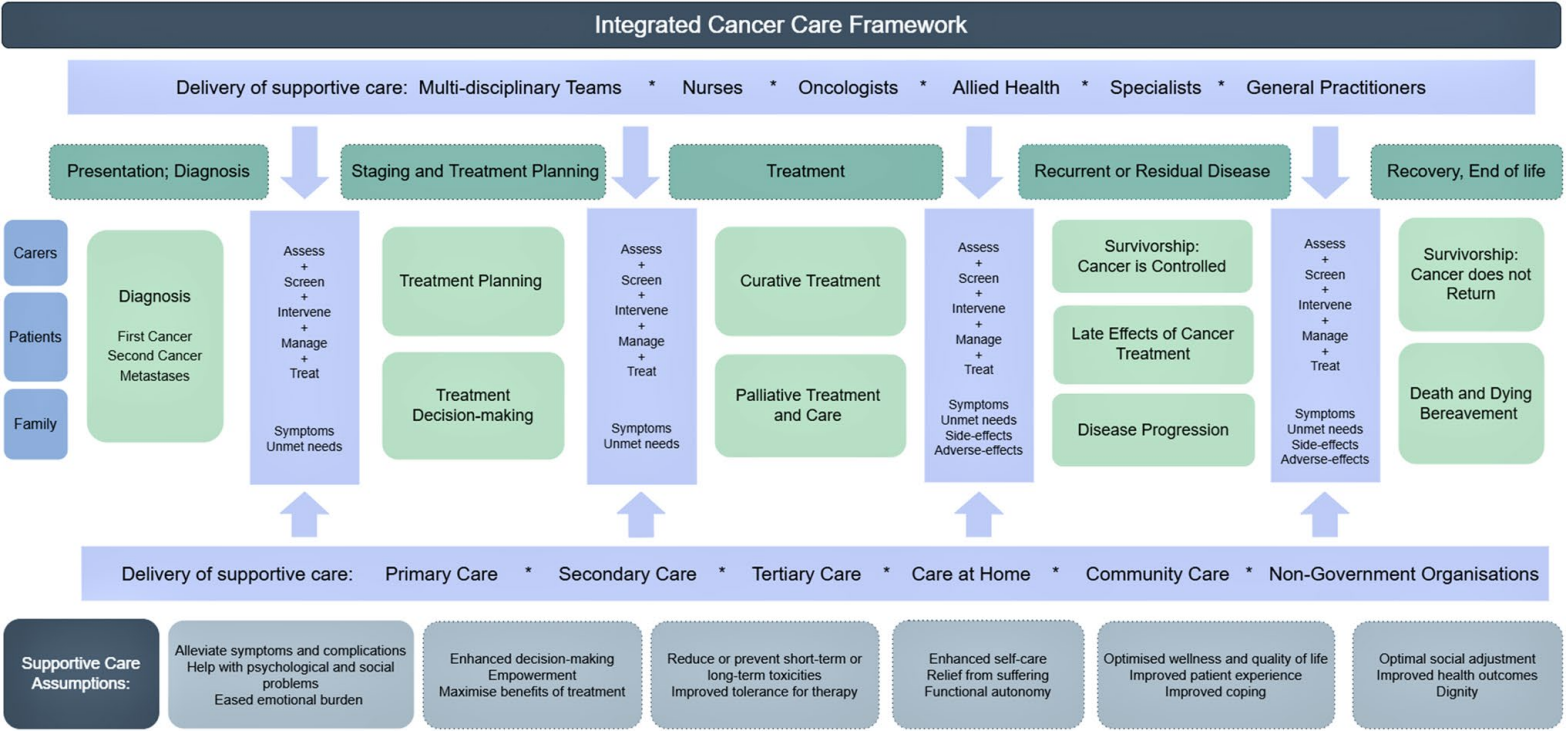
Integrated cancer care framework

Terms and categories which met consensus were assessed using ToC to map provision of cancer supportive care as a complex intervention [19]. Consensus terms and categories listed above were analysed to determine the inputs and outputs necessary for provision of supportive care, and to make explicit the multiple causal pathways and feedback loops between service provision and the intended outcomes of having supportive care integrated as a platform underpinning delivery of cancer services (Fig. 1). The statements of definition and the conceptual framework were presented to participants in the Delphi round 2 survey.

**Table:** 

**Statements of definition: Cancer Supportive Care**
*What* Supportive care is a core, evidence-based component of comprehensive patient-centred cancer care *Who and When and Where* The delivery of supportive care requires a multi-disciplinary approach, in the screening, assessment, management, intervention and treatment of the side-effects, symptoms and needs of cancer patients, carers and family. Supportive care is delivered in all healthcare settings, at all steps of the cancer pathway from diagnosis to survivorship and end of life *Why* Importantly, the provision of supportive care is designed to: empower and enhance decision-making; maximise tolerance and benefit from therapy; alleviate symptoms of cancer and side-effects of treatment; optimise functional autonomy, wellness and health outcomes; and improve coping, self-care and dignity

### Delphi round 2 survey

A total of 37 respondents took part in the second round of the Delphi, with response rate of 67% from consent (Table 2). Most participants strongly agreed/agreed/somewhat agreed with all 11 statements presented about the statements of definition and framework. Importantly, respondents felt that the definition statements are effective in conveying what cancer supportive care entails (*n* = 36, 97%), are meaningful and useful to guide best practice models (*n* = 34, 94%), and will inform health services of what implementation of comprehensive cancer supportive care requires (*n* = 34, 94%) (Table 3). Similarly, most participants agreed that the framework contained all components of supportive care (*n* = 34, 92%), would be useful in strengthening the evidence-base for integration of cancer supportive care (*n* = 32, 86%), and could help inform health system planning (*n* = 36, 97%) (Table 4).

**Table 3:** Respondent’s agreement with the with the meaningfulness and usefulness of the refreshed concept statements

ITEMS	Strongly agree		Agree		Somewhat agree		Neither agree nor disagree		Somewhat disagree		Disagree		Strongly disagree	
	*n*	%	*n*	%	*n*	%	*n*	%	*n*	%	*n*	%	*n*	%
1. The concept statements reflect the nuances, complexities, and information required to effectively convey what cancer supportive care entails or aspires to achieve (*n* = 37)	14	38	20	54	2	5	0	0	0	0	1	3	0	0
2. The concept statements are meaningful and useful to guide best practice models of cancer supportive care (*n* = 36)	14	39	19	53	1	3	0	0	1	3	1	3	0	0
3. The concept statements are meaningful and useful to help inform health services of what implementation of comprehensive supportive care requires (*n* = 37)	6	16	17	46	10	27	2	5	1	3	0	0	1	3
4. The concept statements are meaningful and useful to help health services assess quality of their supportive care provision (*n* = 37)	6	16	18	49	9	24	2	5	1	3	1	3	0	0
5. The concept statements are meaningful and useful to guide future research endeavours (*n* = 36)	11	31	13	36	8	22	3	8	1	3	0	0	0	0
6. The concept statements are meaningful and useful to convey/explain supportive care to patients, carers and family members (*n* = 37)	9	24	17	46	6	16	0	0	2	5	3	8	0	0
7. The concept statements are meaningful and useful to convey/explain supportive care to multidisciplinary clinicians (*n* = 36)	15	42	12	33	5	14	2	6	1	3	1	3	0	0

**Table 4:** Respondent’s agreement with the usefulness of the proposed framework to strengthen research, health system planning, and integration

	Strongly agree		Agree		Somewhat agree		Neither agree nor disagree		Somewhat disagree		Disagree		Strongly disagree	
1. The framework contains all essential/core components of cancer supportive care	8	22	21	57	5	14	0	0	2	5	1	3	0	0
2. The framework can guide or inform future research direction to strengthen the evidence base for integration of cancer supportive care	9	24	18	49	5	14	2	5	2	5	1	3	0	0
3. The framework can help inform health service or system planning for integration of supportive care into cancer treatment/care	8	22	22	59	6	16	0	0	0	0	1	3	0	0
4. The framework clearly demonstrates how and where supportive care integrates within the health system to facilitate comprehensive cancer care	9	24	21	57	3	8	1	3	2	5	1	3	0	0

### Open text comments

The small proportion of participants who selected ‘somewhat disagree/disagree/strongly disagree’ with the 11 statements about the statements of definition and framework were invited to provide additional comments regarding their selection. Predominantly, disagreement was due to perceived gaps in detail. For example, ‘more detail with regard to who delivers supportive care’ was reported by one participant as the reason why they disagreed with statement 3, describing usefulness to inform health services regarding implementation of comprehensive cancer supportive care. Statement 6 had the most disagreement, with five participants stating they felt that the statements may be too complex for patients and their family. A separate patient or ‘lay’ version of the definitions of supportive care was suggested. One participant who disagreed with statement 3 did not give a reason for this, and for statement 4, comments were provided that referred to disparities in healthcare access rather than responding about the framework itself.

## Discussion

Our study set out to explore the need for a refreshed supportive care definition and framework to refocus the dialogue about cancer supportive care. Refreshed statements of definition were generated and endorsed by international experts in cancer supportive care. These contributions were provided by predominantly female participants—reflective of the workforce and those mainly involved in provision of supportive care. The statements align strongly with international policy and advocacy documents that focus on early identification, timely intervention, multidisciplinary collaboration, and end-to-end, cross-sector care—characteristics that cannot equitably or effectively be achieved through an individual-level or fragmented approach to care provision [7, 8, 26].

While cancer supportive care has long been recognised as an important component of cancer service delivery [6–8], published evidence suggests an ongoing, high burden of unaddressed need across all supportive care domains for many patient groups, at all stages of their cancer experience. In part, these data have been explained by inadequacy of or inability to resource integration of supportive care, into routine cancer services. Our study findings suggest that a focus on investment, although important, may overlook a critical issue. That is, that supportive care is much more than a series of discreet services that co-occur within cancer services, but rather is a conceptual framework guiding the planning, resourcing, and delivery of cancer care. Adoption of a supply or service-driven approach to cancer supportive care has consistently failed to demonstrate value to patients or health systems and has resulted in disinvestment in delivery of comprehensive supportive care [17].

Since the publication of Fitch’s original supportive care framework in 2008, there has been a revolution in cancer treatments. People are living longer with the consequences of cancer and cancer therapy, and health service use has grown exponentially. As people live longer with the consequences of cancer and cancer treatments, the impact of insufficient or inaccessible supportive care and the challenges this presents for recovery is increasingly apparent. Subsequently, insights provided by those affected by cancer contribute important understanding of where and why improvement is needed.

As such, identifying, intervening, preventing, or mitigating the myriad consequences of cancer has become a value-based proposition for cancer care providers looking to effectively and efficiently, use increasingly scarce resources. Our work asserts that there is pressing need to refocus cancer supportive care. Our refreshed integrated cancer care framework, developed and endorsed through consensus by international experts, re-orients the conversation about cancer supportive care from a discussion about service delivery and discrete interventions to a value-based health system frame of reference concerned with reducing fragmentation and achieving outcomes that matter to patients. This assertion refocuses supportive care to Fitch’s original framework and intent.

Our refreshed statements and framework support a shift in thinking away from the current understanding of supportive care to an appreciation of its importance as the basis for delivery of integrated cancer care [14, 29, 30]. Unlike existing international models of supportive care, where supportive care is articulated as one component of cancer care [6, 7, 26], our framework offers a new paradigm for integrated cancer care, where each component of cancer care and treatment occur within a supportive care framework. Delivery of treatments is no longer conceptualised as the primary activity of cancer care, where all other aspects of care are additive or recommended, but rather, delivery of cancer therapies or excellent symptom management is understood to optimally occur within a framework of supportive care. It offers a way of clarifying the blurred lines between supportive, palliative, end of life, and survivorship care, demonstrating that the delivery of acute cancer care, the disciplines of survivorship, palliative, and end of life care interconnect with each other within a supportive care frame of reference. Importantly, this conceptualisation is much more aligned with how people experience their lives, where there is integration of the various domains (separated largely for our convenience as health care professionals and researchers, for description and measurement), but which can be very challenging for individuals to articulate as discrete events or issues. Persisting with outdated models, where supportive care sits alongside other aspects of cancer care, will further the misconception that the provision of supportive care is additive to cancer care delivery, rather than the premise upon which all cancer care is delivered [6–8].

At an individual patient level, the refreshed statements and framework facilitate identification of patient needs at a population and individual level. At the health service level, they provide opportunity to explore improved healthcare system performance, cost effectiveness of integrated cancer care, and demonstration of improvement through use of contemporary quality indicators. At a clinical level, the framework and quality indicators provide opportunity to better understand workforce training needs and skills requirements, and better enable individual clinicians to understand their role within the multi-disciplinary application of integrated cancer care.

Supportive care has frequently been misunderstood as an optional or non-essential aspect of cancer care [7]. Our framework offers a refocused and refreshed proposition—radical in its conceptualisation—–which proposes an understanding of cancer supportive care as the template for integrated cancer service planning and delivery. It shifts an understanding of supportive care as a sub-speciality, discipline, or series of interventions to a way of understanding, planning, delivering, and evaluating integrated cancer care.

Underpinned by a series of supportive care assumptions developed through the use of Theory of Change, the framework offers insight to meaningful outcomes for supportive cancer care research that focus on maximising health outcomes that matter to people affected by cancer and the system within which care is delivered. Our work contributes new perspectives to the literature on supportive care. It offers health service administrators, policy makers, health services researchers, and multidisciplinary clinicians an opportunity to re-envision supportive care as a conceptual framework to plan, deliver, and evaluate quality cancer care. Importantly, our work orients supportive care as the fundamental context through which all other aspects of cancer care are delivered.

## Data Availability

Available upon request.
